# Saliva—Friend and Foe in the COVID-19 Outbreak

**DOI:** 10.3390/diagnostics10050290

**Published:** 2020-05-09

**Authors:** Pingping Han, Sašo Ivanovski

**Affiliations:** School of Dentistry, The University of Queensland, Herston, QLD 4006, Australia; p.han@uq.edu.au

**Keywords:** COVID-19, salivary diagnostics, salivary bioaerosols transmission

## Abstract

The coronavirus disease 2019 (COVID-19) outbreak, caused by the novel severe acute respiratory syndrome coronavirus 2 (SARS-CoV-2), has become a global ongoing pandemic. Timely, accurate and non-invasive SARS-CoV-2 detection in both symptomatic and asymptomatic patients, as well as determination of their immune status, will facilitate effective large-scale pandemic control measures to prevent the spread of COVID-19. Saliva is a biofluid whose anatomical source and location is of particularly strategic relevance to COVID-19 transmission and monitoring. This review focuses on the role of saliva as both a foe (a common mode of viral transmission via salivary droplets and potentially aerosols) and a friend (as a non-invasive diagnostic tool for viral detection and immune status surveillance) in combating COVID-19.

## 1. COVID-19 Pandemic 

Coronavirus disease 2019 (COVID-19) is a highly prevalent, acute infectious respiratory disease that is caused by the novel severe acute respiratory syndrome coronavirus 2 (SARS-CoV-2) virus. It has resulted in major disruption to the everyday life of a majority of the world’s population by significantly disrupting public health, and the social and economic fabric of society. 

This disease was initially detected in the city of Wuhan, China, in December 2019, and has subsequently become a global pandemic. COVID-19 is considered as the 6th public health emergency of international concern by the World Health Organisation (WHO) [1]. At the time of preparation of this review (21 April), >2.4 million laboratory-confirmed cases and >170,000 deaths have been reported globally from 213 countries, areas, or territories [2,3]. The reported case fatality rate (CFR) varies dramatically between different countries, from 12.73% in Italy to 0.4% in New Zealand [4], and poses an escalating public health emergency.

Current clinical studies show that common respiratory symptoms of COVID-19 include fever, fatigue, dry cough, and shortness of breath, which can progress to severe viral pneumonia and multi-organ failure in susceptive patients [5]. Other symptoms, including anosmia (loss of smell) and ageusia (loss of taste), have also been detected among confirmed cases [6]. Asymptomatic patients are largely overlooked in the current diagnosis strategies of many countries. However, they may serve as a reservoir and contribute to the spread of the disease, making up a reported 30% of cases in South Korea [7], 17.9% on the Diamond Princess cruise ship [8,9], 60% on the Greg Mortimer cruise ship [10], and 50%–75% in Italy [11]. The overall prevalence of asymptomatic patients, and the mechanism of how they drive this pandemic, remain unknown. In response to the COVID-19 pandemic, early, accurate, and sensitive diagnosis for both symptomatic and asymptomatic patients may provide efficient and timely disease control, particularly in identifying potential “super-spreaders”. 

This review explores the potential role of saliva in the COVID-19 pandemic, as both a mechanism for the spread of the disease and a readily accessible diagnostic tool for detecting the presence of the virus, as well as an individual’s immune status.

## 2. What is SARS-CoV-2?

According to currently available genome sequencing data, SARS-CoV-2 is a novel zoonotic enveloped positive-sense single-stranded RNA virus from the Coronaviridae family that was identified in the 1960s [12]. SARS-CoV-2 shares 96% identity with a bat coronavirus (BatCoV-RaTG13) [13], 91.02% with pangolin-CoV [14], and 79.5% with severe acute respiratory syndrome coronavirus (SARS-CoV) [15], respectively, at the whole-genome level. The original severe acute respiratory syndrome coronavirus (SARS-CoV) [16], Middle-East respiratory syndrome coronavirus (MERS-CoV) [17], and SARS-CoV-2 belong to a β-coronavirus genus which infects mammals and humans. SARS-CoV emerged in China in November 2002; the SARS epidemic ended abruptly in July 2003, with no human SARS cases detected since 2004 [16]. Although both SARS-CoV and SARS-CoV-2 originated from, and are closely related with, bat coronavirus, whether the SARS-CoV-2 has an intermediate host remains unknown. Additional sequencing data from other wild animals and mammals are required to confirm the source and origin of SARS-CoV-2.

**Figure 1:**
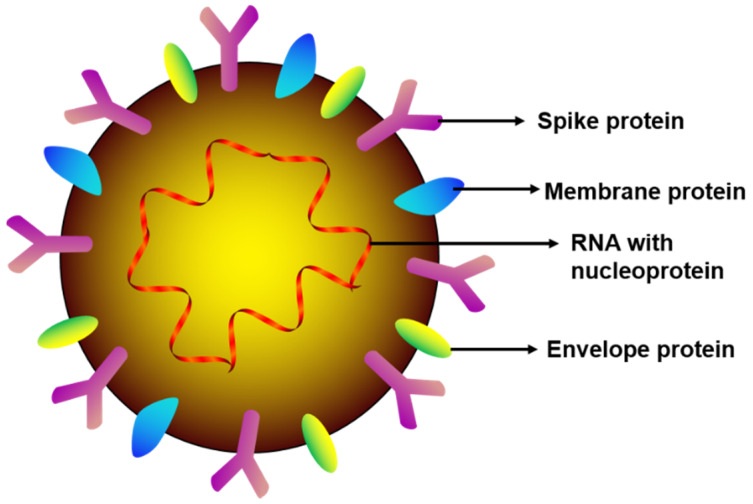
Schematic showing the SARS-CoV-2 virus structure, with spike (S), membrane (M), envelope (E), and nucleocapsid (N) proteins and single-stranded RNA genome.

According to the cryogenic electron microscopy (TEM) images, the SARS-CoV-2 virion is crown-shaped with a diameter of ~50–200 nm [18], having four structural proteins: spike (S), envelope (E), membrane (M), and nucleocapsid (N) (illustrated in Figure 1). The S, E, and M proteins are responsible for viral envelope generation and the N protein carries the RNA genome (~30 kb). Of note, the spike protein is the glycoprotein that facilitates SARS-CoV-2 attachment, fusion, entry, and transmission into host cells by binding with human angiotensin converting enzyme 2 (hACE2) receptors [19], which are expressed by epithelial cells of the lung, intestine, kidney, blood vessels, and oral mucosa [20]. The detailed mechanism of how SARS-CoV-2 S protein binds with hACEs, and ultimately leads to pathological organ damage, remains unknown and requires further investigation. 

## 3. Transmission of COVID-19

While the modes of SARS-CoV-2 spreading are still being investigated, human-to-human airborne transmission of the virus has been confirmed during breathing, coughing, sneezing, and conversing in close contact (1–3 metres). Airborne transmission of the virus appears to be a primary mode for the spread of COVID-19, with positive viral RNA detected in air samples between two isolated patients (>1.8 metres distancing), as well as in air samples outside patients’ isolation rooms [21]. The extent to which SARS-CoV-2 virus can travel over longer distances is currently unknown [22], although anecdotal evidence from the rapid and widespread spread in environments such as cruise ships, where people have been confined to their cabins and practice hand hygiene, suggest that the virus can travel over longer distances possibly via internal ventilation systems. 

The SARS-CoV-2 virus can survive on a variety of surfaces, including on plastic for 72 h, on stainless steel for 48 h, on copper for 8 h, cardboard after 24 h [23], and on a surgical mask for 7 days [24], subject to favourable humidity and temperature. Like other coronaviruses, SARS-CoV-2 virus can be stored at −80 °C for several years and inactivated at 56 °C for 30 min. Additionally, 75% ethanol, 0.1% sodium hypochlorite, and 0.5% hydrogen peroxide can inactivate SARS-CoV-2 [25]. 

The incubation period in susceptible COVID-19 patients is 1–14 days, with an average of 3–7 days [18]. From the existing data, the SARS-CoV-2 virus can be detected in multiple sources, including gastrointestinal tissue [26], tears [27], stool [28], blood [29], and saliva [30,31,32,33] of COVID-19 patients. 

Initial mathematic modelling suggests that the basic reproductive number (R_0_) of SARS-CoV-2 is expected to be 1.4–3.9 [34], indicating that one infection would lead to 1.4 to 3.9 new infections with no interventions; where R_0_ estimates may vary upon biological, social-behavioural and environment factors [35]. 

## 4. Current COVID-19 Diagnosis 

Rapid identification and publication of the virus’ genome sequence have facilitated the development of diagnostic methods, as well as the race to develop a vaccine. The standard method of COVID-19 detection is reverse transcription polymerase chain reaction (RT-qPCR), generally used to detect viral RNA from nasopharyngeal and oropharyngeal swabs or sputum samples. Qualitative reverse transcription polymerase chain reaction (RT-qPCR) assays are easier to validate than quantitative assays and are preferred for diagnostics. Furthermore, a chest X-ray could be a useful diagnostic tool to detect bilateral pneumonia, presenting as multilobar ground-glass opacities with a peripheral, asymmetric, and posterior distribution [36]. 

Alarmingly, some healthcare patients remain viral RNA positive 13 days after hospital discharge and may even relapse [37], suggesting a virus-eliminating immune response to SARS-CoV-2 may not occur in some patients. As of 19 March 2020, a serology antibody test to detect immunoglobulin G (IgG) and IgM was approved by the FDA as a point-of-care test, though is not yet widely used. It is likely that as the pandemic reaches the next phases, increased focus will be placed on monitoring immunity within the population.

## 5. Salivary Droplets and Bioaerosols: A Hidden Foe in COVID-19 

Airborne transmission of viruses can generally occur in two ways: either through relatively large droplets of respiratory fluid (10–100 μm) or through smaller particles called aerosols (<10 μm). The larger droplets are pulled to the ground by gravity quickly and hence transmission requires close physical proximity, whereas aerosolised transmission may occur over larger distances and does not necessarily require infected and susceptible individuals to be co-located at the same time [38]. Respiratory and salivary droplets appear to be the main transmission routes of COVID-19 disease through inhalation, ingestion, and/or direct mucous contact [39]. Indeed, it has been suggested that such droplets can travel up to four metres with an uncovered cough [40]. It has also been shown that the SARS-CoV-2 virus can survive in aerosols in an experimental setting [23,24], but it is unclear to what such particles are generated in “real-life” situations, and whether such particles are sufficient to cause an infection. Therefore, the aerosol route for COVID-19 transmission requires further verification in clinical settings, taking into account the presence of patients and health workers, air circulation and other environmental factors. 

The potential for transmission via salivary bioaerosols poses a particularly significant danger to healthcare workers that operate in close proximity to the face and oral cavities, such as dental practitioners; oral-maxillofacial surgeons; ear, nose, and throat (ENT; otorhinolaryngology) surgeons; and ophthalmologists, especially when carrying out procedures that generate aerosols [41,42]. Indeed, the COVID-19 outbreak has resulted in the significant curtailment of services provided by these health professionals, posing a significant public health problem, as important and highly prevalent oral and ENT conditions cannot be adequately treated during this epidemic [41,42,43,44]. Thus, understanding the role of salivary aerosols in COVID-19 transmission is imperative, as is an appreciation of the effect of various environmental and therapeutic interventions on the extent of aerosol creation, and the development of strategies to minimise the risk to both health professionals and patients alike. 

The role of pre-procedural rinsing [45] with disinfectant mouthwash needs to be explored in this context. Similarly, the use of personal protective equipment, such as masks and respirators which could be effective in preventing the airborne transmission of coronavirus RNA [46], needs to be tested in clinically relevant situations where droplets and aerosols are generated from biofluids (including saliva) during medical procedures. Similarly, high volume suction and use of filtration air-systems, especially in clinical settings where aerosols (including those from saliva) can be generated by surgical procedures, requires further investigation.

## 6. Salivary Diagnosis and Monitoring: The Friends in Combating COVID-19

### 6.1. The Versatility of Saliva for COVID-19 Diagnosis 

The timing (highest viral titres) and specimen collection sources can significantly influence the diagnostic sensitivity of SARS-CoV-2 detection tests. One study reported that oropharyngeal swabs (*n* = 398) were more often used than nasopharyngeal swabs (*n* = 8) in China during the COVID19 outbreak; however, SARS-CoV-2 RNA was detected in only 32% of oropharyngeal swabs [47]. On 19 March 2020, the World Health Organisation (WHO) recommended that both upper (nasopharyngeal and oropharyngeal swabs) and lower (sputum, bronchoalveolar, or lavage endotracheal aspirate) respiratory specimens should be collected; however, upper respiratory samples may fail to detect early viral infection and the collection of lower respiratory specimens increases biosafety risk to healthcare workers via aerosol/droplets formation. As the SARS-CoV-2 virus shedding progresses, additional samples sources, such as stool, saliva, and blood, can be used as alternatives, or combined with respiratory specimens. However, only 15% of patients hospitalised with pneumonia had detectable SARS-CoV-2 RNA in serum [48], and 55% of patients showed positive SARS-CoV-2 RNA in fecal samples [49]. Conversely, in saliva samples, it was reported from different clinical studies that 87%, 91.6%, and 100% of COVID-19 patients were identified as being viral positive, respectively [30,31,33], suggesting that saliva is a powerful specimen source for the diagnosis of the SARS-CoV-2 virus.

**Figure 2:**
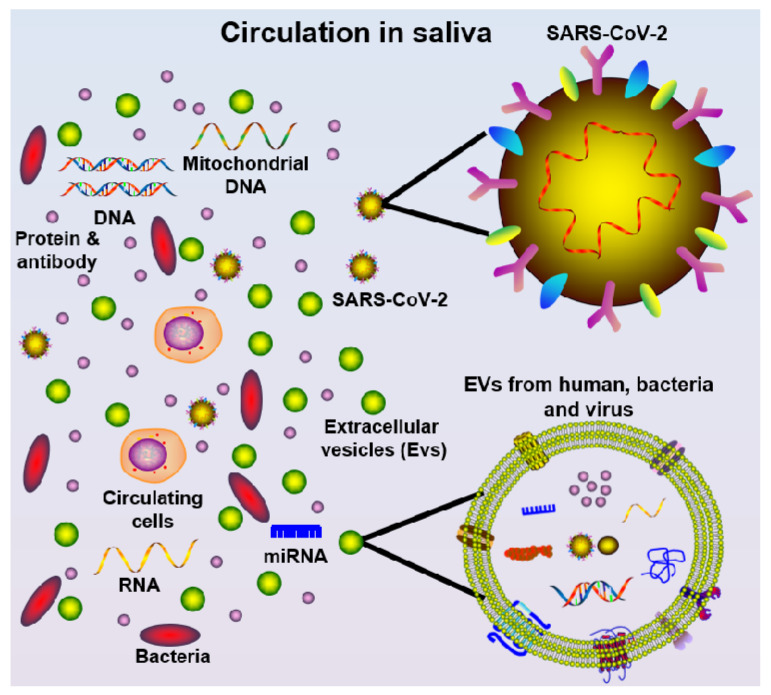
Schematic diagram of saliva components, including cells, mitochondrial DNA, DNA, protein/antibody, bacteria, miRNA, extracellular vesicles (EVs, from multiple oral cavity resident species), and SARS-CoV-2 virus.

Saliva also represents an attractive biofluid source option for the detection of SARS-CoV-2, due to being non-invasive, easy-to-access, and low-cost, as well as having the ability to “mirror” systemic and local disease status [50]. It is well-known that saliva harbors a wide range of circulatory components (Figure 2), such as pro-inflammatory cytokines [51,52], chemokines [53], matrix metalloproteinases [54,55], mitochondrial DNA [56], genomic DNA [57], bacteria [58], SARS-CoV and SARS-CoV-2 virus [30,31,59], SARS-CoV antibodies [59], miRNAs [60], and extracellular vesicles (EVs) [61]. Furthermore, saliva samples can be stored at –80 °C for several years with little degradation [62]. It is preferable to aliquot and freeze the samples to avoid freeze–thaw cycles. For salivary RNA research, it was discovered that saliva samples can be stored in Trizol for more than two years at –80 °C without adding RNase inhibitors [63,64], suggesting such specimens can be used for future diagnostics. Thus, saliva may be a valuable specimen to collect in COVID-19 patients at different time points during disease onset progression and follow-up. Indeed, saliva may be useful for both diagnosing the presence and sequelae of COVID-19 infection, as well as identifying and tracking the development of immunity to the virus.

### 6.2. Salivary Diagnostics for COVID-19

Saliva has been widely investigated as a potential diagnostic tool for chronic systemic and local (oral) diseases [50], with less attention given to its utility in acute infectious diseases, such as COVID-19. The salivary gland can be infected by SARS-CoV-2 virus resulting in the subsequent release of viral particles or antibodies into saliva, as evidenced in Rhesus macaque primates where salivary gland epithelial cells were the first target cells for SARS-CoV infection [59]. This is likely to be facilitated by the high expression of hACE2 (SARS-CoV-2 receptor) on the epithelial cells of the oral mucosa, as demonstrated using single-cell RNA sequencing [65]. 

Saliva and throat wash (by gargling 10 mL saline) samples from 17 SARS-CoV patients were found to be SARS-CoV RNA positive, with the highest detection rate a median of four days after disease onset and during lung lesion development [66]. Saliva samples from 75 patients successfully validated saliva as a viable biosample source for COVID-19 detection when compared to nasopharyngeal or oropharyngeal swabs [67]. 

At present, only three clinical studies (Table 1) and one animal model have investigated the use of salivary diagnostics for COVID-19. SARS-CoV-2 was detected in self-collected saliva (by asking the patients to expectorate saliva) in 11 out of 12 confirmed cases [31]. Another recent study found that 100% of COVID-19 patients (*n* = 25) were detected as viral positive in drooling saliva samples [33]. Further, in a cohort of COVID-19 positive patients, it has been demonstrated that 87% of posterior oropharyngeal (deep throat) saliva samples were detected viral positive (*n* = 23), and serial respiratory viral load of SARS-CoV-2 was detected from week 1 and up to 25 days after symptom onset, while serum (*n* = 16) samples showed positive RT-qPCR detection only 14 days after symptom onset [30]. Additionally, Kim et al. demonstrated that SARS-CoV-2-infected ferret animals shed virus in nasal washes, saliva, urine, and feces up to eight days post-infection and ferret-to-ferret transmission occurred only two days post-contact [32]. Notwithstanding the limitations of small sample size and lack of detailed saliva collection methodology, these studies nevertheless imply that saliva is a promising non-invasive alternative specimen for SARS-CoV-2 diagnosis. Further investigations are required to explore the potential role of saliva for COVID-19 detection in both symptomatic and asymptomatic patients.

In summary, the current gold standard diagnostic test is RT-qPCR to detect SARS-CoV-2 RNA which takes approximately 48 h to obtain the test results. More new tests with higher sensitivity and specificity need to be appropriately validated before being implemented into the current routine diagnosis.

### 6.3. Salivary Immunity Monitoring for COVID-19

From early reports on the clinical characteristics of COVID-19, it is now apparent that not all people exposed to SARS-CoV-2 are infected and not all infected patients develop severe symptoms [18]. Indeed, three broad presentations of SARS-CoV-2 infection can be characterised: (i) an asymptomatic incubation stage with or without detectable virus; (ii) non-severe symptomatic presentation with confirmed presence of virus; and (iii) a severe respiratory symptomatic stage with high viral load [68]. Determining the immune status of an individual is likely to become increasingly critical as the COVID-19 pandemic progresses, because from a prevention perspective, individuals at stage I (the stealth carriers or the super spreaders), are particularly important because they may spread the virus unknowingly.

Two stages of the immune response during COVID-19 disease progression have been proposed [69]: (1) Immune-defense-based protective phase: elimination of SARS-CoV-2 virus by an individual’s adaptive immune response; and (2) inflammation-driven phase: when the protective immune response is impaired and prolonged propagated virus load leads to an adverse inflammatory response in organs with high hACEs expression. Indeed, a likely pathogenic mechanism of SARS-CoV-2 is overactivation of T cells with an increase in CD4+ T Helper cells and enhanced cytotoxicity of CD4+ and CD8+ T cells [70], which leads to an imbalanced pro-inflammatory and anti-inflammatory cytokine response and severe immune injury in susceptible patients [71]. Although this concept needs to be confirmed by more clinical research, it may provide useful research directions to tackle COVID-19. 

During the previous SARS outbreak, a common transmission pattern hypothesis was that SARS-CoV virus silently infected asymptomatic patients, which may have led to population immunity against infection (herd immunity) that may explain the eradication of the virus, although this is yet to be confirmed [72]. Although a study suggests that coronavirus antibodies are highly prevalent in the general population after exposure to four non-SARS coronavirus strains [73], there is no definitive evidence on whether permanent immunity would be generated against other CoV species, such as SARS-COV-2. Notably, after SARS-CoV infection in a murine model, the production of SARS-CoV-specific serum IgG and secretory immunoglobulin A (sIgA) were detected in saliva following intranasal immunisation [74]. 

In relation to COVID-19, intensive care unit (ICU) patients had higher plasma levels of pro-inflammatory cytokines, including IL-2, IL-7, IL-10, GSCF, IP10, MCP-1, MIP-1A, and TNF-α, compared with non-ICU patients [48], suggesting the emergence of a robust immune-inflammatory response in severe symptomatic COVID-19 patients. Importantly, several studies have demonstrated that COVID-19 patients developed IgG and IgM antibodies against SARS-CoV-2 in blood samples. Both IgG and IgM antibodies against the SARS-CoV-2 nucleoprotein and spike receptor-binding domain were increased in serum at day 10 after symptom onset for up to three weeks [30]. A point-of-care lateral flow immunoassay (LFIA) test product (VivaDiag COVID-19 IgM/IgG Rapid Test) was designed to detect IgM and IgG in blood samples of COVID-19 patients in 15 min [75]. However, the sensitivity of the VivaDiag COVID-19 IgM/IgG Rapid Test was only 18.4% in blood samples of acute COVID-19 patients from the emergency department [76], suggesting that serological tests require more research before being deemed suitable for routine diagnosis. Additionally, the seroconversion rate for total antibodies, IgM, and IgG were shown to be 93.1%, 82.7%, and 64.7%, respectively, in hospitalised COVID-19 patients, peaking 7–14 days after symptom onset [77]. Given the non-invasive and cost-effective nature of saliva collection, it would be important to investigate whether this immunity detection is feasible in saliva samples as a tool for facilitating the testing of COVID-19 immunity at the population-level. 

## 7. Summary: Saliva as Friend and Foe

SARS-CoV-2 is present in saliva by entering the oral cavity through several routes, including direct infection of oral mucosa lining cells, via droplets from the respiratory tract, from the blood circulation through gingival crevicular fluid, or by extracellular vesicles secreted from infected cells and tissues, as described in [43]. As such, saliva is a common route for the transmission of the virus, including airborne transmission via routine activity such as speaking and sneezing, as well as infection-associated symptoms such as sneezing and coughing. Transmission via saliva may represent a particular threat to health workers who work in close proximity to, and undertake procedures within, the oral cavity.

**Figure 3:**
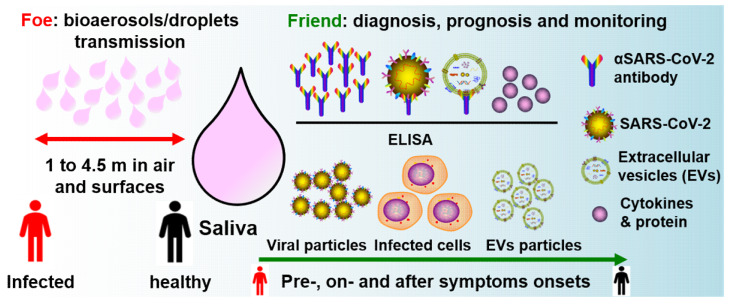
Saliva as a friend and foe in the fight against COVID-19. Saliva has a role in human-to-human transmission via bioaerosols and droplets. Salivary proteins and anti-SARS-CoV-2 antibodies, viral particles, EVs, and infected host cells can be potential diagnostic, prognostic, and COVID immunity monitoring biomarkers, for both symptomatic and asymptomatic patients. EVs: extracellular vesicles; ELISA: enzyme-linked immunosorbent assay.

Aside from salivary viral RNA testing by RT-qPCR, we propose that salivary ELISA of IgM/IgG against SARS-CoV-2, SARS-CoV-2 double-membrane extracellular vesicles (EVs) isolation, anti-SARS-CoV-2 surface proteins, viral titres load, CD4+/CD8+ T cells derived EVs, and pro-inflammatory cytokines could be potential diagnostic and prognostic biomarkers for COVID-19 disease (Figure 3). A salivary test would be particularly important for improving the effectiveness and efficiency of prevention strategies for healthcare professionals, especially when performing aerosol-related procedures. Indeed, an ideal saliva test would be a disposable “off-the-shelf” device that could be used at home by individuals, without exposing them or others to a potential environmental virus infection risk.

In conclusion, although saliva is currently perceived as a foe in the battle against COVID-19 due to it being a prominent source for disease transmission via droplets and possibly aerosols, it is also apparent that it can be harnessed as a friend in the detection of the virus and an individual’s immunity to it. Indeed, non-invasive saliva sampling may be an alternative cost-effective method for improving the sensitivity and accuracy of large-scale detection of COVID-19 virus and/or immunity, hence significantly decreasing the risk for medical professionals and patients. 

## Figures and Tables

**Table 1:** Current clinical research finding using salivary diagnosis for COVID-19.

	Sample Size Age (Years)	Sample Source	Diagnosis Technique	Diagnosis Efficiency in Saliva	Reference
To et al.	10 Female, 13 Male Median: 62 (37–75)	Posterior oropharyngeal saliva	RT-qPCR	87% of patients were viral positive	[30]
To et al.	5 Female, 7 Male Median: 62.5 (35–75)	Saliva from throat	RT-qPCR	91.7% of patients were positive	[31]
Azzi et al.	8 Female, 17 Male Mean ± standard deviation: 61.5 ± 11.2	Drooling saliva	RT-qPCR	100% of patients were viral positive	[33]
